# Human Umbilical Cord Mesenchymal Stem Cell-Derived Exosomes Attenuate Oxygen-Glucose Deprivation/Reperfusion-Induced Microglial Pyroptosis by Promoting FOXO3a-Dependent Mitophagy

**DOI:** 10.1155/2021/6219715

**Published:** 2021-11-02

**Authors:** Zhenzhen Hu, Ya Yuan, Xiuli Zhang, Yifeng Lu, Na Dong, Xiuqin Jiang, Jinjin Xu, Datong Zheng

**Affiliations:** ^1^Clinical Molecular Diagnostic Laboratory, The Second Affiliated Hospital of Nanjing Medical University, Nanjing, Jiangsu 210003, China; ^2^The Second Clinical Medical School of Nanjing Medical University, Nanjing, Jiangsu 210011, China; ^3^Children's Health Center, The Second Affiliated Hospital of Nanjing Medical University, Nanjing, Jiangsu 210003, China

## Abstract

**Background:**

Mesenchymal stem cell-derived exosomes (MSC-exos) have been recognized as a promising therapeutic strategy for neonatal hypoxic-ischemic brain damage (HIBD). Recently, microglial pyroptosis was shown to play a vital role in the progression of neonatal HIBD. However, whether MSC-exos improve HIBD by regulating microglial pyroptosis remains unknown.

**Methods:**

Exosomes were isolated from human umbilical cord mesenchymal stem cells (huMSCs) and identified by transmission electron microscopy (TEM), western blot, and nanoparticle tracking analysis (NTA). BV-2 cells were subjected to oxygen-glucose deprivation/reoxygenation (OGD/R) to induce microglial ischemia/reperfusion (I/R) *in vitro*. CCK-8, ELISA, western blot, and Hoechst 33342/PI double staining were performed to detect the pyroptosis of BV-2 cells. Conditioned medium (CM) from BV-2 cells exposed to different treatments was used to investigate its effect on neuronal injury. Moreover, 3-methyladenine (3-MA) and mitochondrial division inhibitor-1 (mdi-1) were used to verify the involvement of mitophagy in the protection of MSC-exos against OGD/R-induced pyroptosis in BV-2 cells. Finally, FOXO3a siRNA was used to investigate the involvement of FOXO3a in MSC-exo-induced mitophagy and pyroptosis inhibition.

**Results:**

Exosomes from huMSCs were successfully extracted. In OGD/R-exposed BV-2 cells, MSC-exos increased cell viability and decreased the expression of NLRP3, cleaved caspase-1, and GSDMD-N as well as the release of IL-1*β* and IL-18. Compared with CM from OGD/R-exposed BV-2 cells treated with PBS, CM from OGD/R-exposed BV-2 cells treated with MSC-exos significantly increased the viability of SH-SY5Y cells and decreased LDH release. MSC-exos also increased the expression of TOM20 and COX IV in OGD/R-exposed BV-2 cells. Additionally, 3-MA and mdi-1 attenuated the inhibition of pyroptosis with MSC-exo treatment. Furthermore, FOXO3a siRNA partially abolished the neuroprotective effect of MSC-exos and attenuated mitophagy and pyroptosis inhibition induced by MSC-exo treatment.

**Conclusions:**

Our findings demonstrated that MSC-exos increased FOXO3a expression to enhance mitophagy, therefore protecting microglia from I/R-induced pyroptosis and alleviating subsequent neuronal injury.

## 1. Introduction

Neonatal hypoxic-ischemic brain damage (HIBD) is not only a serious threat to the lives of newborns but also the most important cause of long-term neurological dysfunction in infants [1, 2]. Currently, only limited therapeutic intervention strategies are available to confer neuroprotective effects in this disease [1–3]. A preliminary clinical study by Kabataş et al. showed that Wharton's jelly-derived mesenchymal stem cells (WJ-MSCs) can effectively improve neurological function and quality of life in a patient with HIBD [4], suggesting that mesenchymal stem cell (MSC) transplantation may be a promising therapy for neonatal HIBD. However, direct application of MSCs is associated with potential risks and hazards, especially in babies who are considered a fragile population [5]. Recently, MSC-derived exosomes (MSC-exos) have been identified as one of the key neuroprotective mechanisms of MSCs and can effectively ameliorate ischemia/reperfusion- (I/R-) induced brain injury by promoting angiogenesis, regulating immune responses, and inhibiting neuronal apoptosis [6–10]. MSC-exos have emerged as an attractive therapeutic alternative that holds great regenerative potential and is cell-free [11]. Therefore, exploring the neuroprotective mechanism of MSC-exos is of great significance to their clinical application.

As the resident immune cells in the central nervous system, microglia play an important role in the occurrence and development of HIBD [12–16]. Previous studies have demonstrated that inhibition of microglial pyroptosis in neonatal HIBD mice could promote neuronal survival and improve brain injury [12, 16]. The key signaling molecules in the classical pyroptosis pathway were found to be increased significantly in the serum of neonates with HIBD and positively correlated with the severity of the disease [16]. The above findings suggest that the reduction in microglial pyroptosis may be the key for curing neonatal HIBD. Recently, MSC-exos were reported to inhibit pyroptosis in a variety of cells [17–19]. However, whether MSC-exos improve neonatal HIBD by inhibiting microglial pyroptosis remains unknown.

Mitophagy, a way to clear damaged mitochondria, plays a vital role in the activation and survival of microglia [20–22]. A recent study has showed that hypercapnia promotes microglial pyroptosis by inhibiting mitophagy in hypoxemic adult rats [23], suggesting that mitophagy acts as a negative regulator of microglial pyroptosis. To date, the effect of mitophagy on I/R-induced microglial pyroptosis remains unclear. Recently, MSC-exos have been shown to exert their protective role by promoting mitophagy in animal models of different diseases, such as nonalcoholic steatohepatitis [24] and cigarette smoke- (CS-) induced lung inflammation [25]. Therefore, it is worthwhile to explore whether mitophagy is involved in the protective effect of MSC-exos against I/R-induced microglial pyroptosis. In the present study, we investigated the effect and mechanism of MSC-exos against I/R-induced microglial pyroptosis *in vitro* using an oxygen-glucose deprivation/reperfusion (OGD/R) model in BV-2 cells.

## 2. Methods

### 2.1. Cell Culture

Human umbilical cord MSCs (huMSCs, cat. DF-GMP-ZB09BA) and the murine microglial BV-2 cell line (cat. ZQ0397) and human neuroblastoma cell line SH-SY5Y (cat. ZQ0050) were obtained from Shanghai Zhong Qiao Xin Zhou Co., Ltd. (Shanghai, China). HuMSCs were cultured in HUMSC complete medium (Zhong Qiao Xin Zhou, cat. ZQ-1320). BV-2 cells were cultured in MEM medium (Zhong Qiao Xin Zhou, cat. ZQ-300) supplemented with 10% fetal bovine serum (FBS, 1693361, Gibco, USA), 100 U/mL penicillin, and 100 U/mL streptomycin. SH-SY5Y cells were cultured in MEM/F12 complete medium (cat. ZQ-1205, Zhong Qiao Xin Zhou). All cells were incubated in a humidified atmosphere of 5% CO_2_ at 37°C.

### 2.2. Exosome Isolation and Characterization

Exosomes were extracted from the cell culture supernatants of huMSCs using a VEX exosome isolation reagent in accordance with the manufacturer's instructions (cat. R601, Vazyme, Nanjing, China). In brief, an initial spin was performed at 3,000 g 4°C for 10 minutes to remove cells, and the supernatant was passed through a 0.22 *μ*m filter (Millipore, Massachusetts, USA) to pelletize and exclude contaminating dead cells and debris. Then, 1/3 of the VEX Exosome Isolation Reagent was added proportionally to the starting sample volume, according to the manufacturer's instructions. Mixtures were vortexed and incubated at 4°C for up to 16 h and then centrifuged at 10,000 g 4°C for 30 minutes to precipitate exosome pellets. Pellets were resuspended in 1× PBS, and the resuspension volume for exosome pellets was 200 *μ*l for 20 mL starting volumes according to the manufacturer's instructions. The bicinchoninic acid (BCA) protein assay kit (cat. BCA1-1KT, Sigma) was used to estimate exosome concentrations. The amount of MSC-exos obtained was 30 *μ*g/mL medium. All exosomes were stored at -80°C immediately after isolation until further analysis. For transmission electron microscopy (JEM-1230, JEOL Ltd., Akishima, Japan), 10 *μ*l of each sample was added to a copper mesh and precipitated for 3 min. The remaining liquid was carefully pipetted from the filter paper edge. Thereafter, the filter paper was rinsed with PBS, and phosphotungstic acid was used for negative staining prior to drying at room temperature for 2 min and imaging (operating voltage: 80-120 kV). The expression levels of exosome-specific biomarkers, CD9, CD63, and Alix, were analyzed by western blot. Nanoparticle tracking analysis (NTA) was performed as previously described [26]. Briefly, the exosome particle size and concentration were measured using NTA at Vivacell Biosciences with ZetaView PMX 110 (Particle Metrix, Meerbusch, Germany) and the corresponding software ZetaView 8.04.02. Isolated exosome samples were appropriately diluted using 1× PBS buffer to measure the particle size and concentration. NTA measurement was recorded and analyzed at 11 positions. The ZetaView system was calibrated using 110 nm polystyrene particles. Temperature was maintained at approximately 23°C and 30°C.

### 2.3. OGD/R Induction, Exosome Treatment, and Collection of Conditioned Medium

For OGD/R induction, BV-2 cells were cultured in glucose-free MEM (cat. PM150443, Procell, Wuhan, China) and then transferred to a sealed hypoxic box containing a mixture of 95% N_2_ and 5% CO_2_ at 37°C for 4 h. Thereafter, the cells were cultured in normal MEM with 10% FBS and maintained for 24 h in reoxygenation under normoxic conditions. BV-2 cells cultured in growth culture medium under normoxic conditions served as a control. After being cultured in oxygen-glucose deprivation (OGD) for 4 h, BV-2 cells were treated with fresh normal MEM with 10% FBS containing 40 *μ*g/mL MSC-exos and placed back in a 5% CO_2_ incubator for 24 h. PBS incubation was set as the control. Conditioned medium (CM) from BV-2 cells with different treatments was collected under sterile conditions followed by centrifugation at 3,000 g at 4°C for 10 minutes to remove cells and then diluted to 1 : 1 with MEM/F12 complete medium for further use. CM from normal cultured BV-2 cells was used as the control CM.

### 2.4. Cell Viability Assay

The Cell Counting Kit-8 (CCK-8, cat. #HY-K0301, MCE, Shanghai, China) assay was used to evaluate the viability of SH-SY5Y and BV-2 cells. The cells were seeded into 96-well plates at a density of 3 × 10^3^ cells/well and then treated as described in the text. Subsequently, 20 *μ*l CCK-8 was added to each well. After 2 h of incubation, the absorbance was measured using a plate reader (ELx800, BioTek Instruments, Inc., Vermont, USA) at 450 nm (A450). Cell viability was calculated as cell viability = OD (treatments)/OD (controls) × 100%.

### 2.5. Cell Apoptosis Assay

SH-SY5Y cells were treated as described in the text, and then, cell apoptosis was detected by using an Annexin V-FITC Apoptosis Detection Kit (cat. C1062M, Beyotime, Nanjing, China) as previously described by us [27]. In brief, 2 × 10^5^ cells were collected and resuspended in 1× binding buffer, followed by double staining with annexin V-FITC and propidium iodide (PI) for 20 min in the dark at room temperature. The stained cells were analyzed by using a FACSCalibur flow cytometer (BD Biosciences, San Jose, CA) with CellQuest software (BD Biosciences). The percentages of early apoptotic cells and late apoptotic cells were calculated.

### 2.6. Exosomal Labeling

PKH26 (cat. MIDI26, Sigma-Aldrich, Saint Louis, USA) was used to label MSC-exos. MSC-exos were incubated with PKH26 for 5 min. The reaction was then stopped with FBS. After washing with medium, PKH26-labeled MSC-exos (40 *μ*g/mL) were added to BV-2 cells and incubated at 37°C for 24 h. The cells were then incubated with DAPI (cat. 0100-20, Southern Biotech, Birmingham, USA) to stain the nuclei and observed via a fluorescence microscope (DM2500, Leica, Wetzlar, Germany).

### 2.7. Intracellular ROS Staining

BV-2 cells were seeded on a coverslip placed in a 6-well plate and treated as described in the text. The cells were incubated for 30 min at 37°C with 5 *μ*M 2′,7′-dichlorofluoresceindiacetate (CM2-DCFHDA) (cat. C2938, Invitrogen, Carlsbad, USA). After treatment, the cells were washed twice with PBS and fixed in 4% formaldehyde for 20 min. After washing with PBS, the cells were stained with DAPI (cat. 0100-20, Southern Biotech) and analyzed by fluorescence microscopy (DM2500, Leica).

### 2.8. Western Blotting

Western blotting assays were performed as previously described by us [27, 28]. The following primary antibodies were obtained from Cell Signaling Technology (Danvers, MA): GSDMD-N (cat. #36425, dilution 1/2000), cleaved caspase-1 (cat. #4199, dilution 1/2000), NLRP3 (cat. #15101, dilution 1/1000), TOMM20 (cat. #42406, dilution 1/2000), COXIV (cat. #4850, dilution 1/2000), FOXO3a (cat. #2497, dilution 1/2000), and GAPDH (cat. #5174, dilution 1/3000). The Gel-Pro image program (Media Cybernetics, Las Vegas, USA) was used to measure the density of bands in a blinded manner. The protein level was expressed as a percentage of GAPDH to generate a relative protein level, and the relative protein level of the control group was expressed as a value of 1 (100%). Then, the protein levels of the other groups were normalized to this value. The obtained values were subjected to statistical analysis.

### 2.9. ELISA

The concentrations of IL-1*β* and IL-18 in the supernatants of BV-2 cells exposed to different treatments were quantified by using IL-1*β* and IL-18 ELISA kits, respectively (IL-1*β* cat. EMC001b.96.2, IL-18 cat. EMC011.96; NeoBioscience, Shenzhen, China) according to the manufacturer's instructions.

### 2.10. Hoechst 33342/PI Staining

The treated BV-2 cells were stained with a Hoechst 33342/PI Double Stain Kit (cat. CA1120, Solarbio, Beijing, China) according to the manufacturer's instructions. Briefly, 2 × 10^4^ cells were trypsinized and resuspended in 1× binding buffer and double-stained with Hoechst 33342 and PI at 4°C for 20 min. Subsequently, the stained cells were observed with a fluorescence microscope (Leica DM2500, Wetzlar, Germany).

### 2.11. Cell Transfection

FOXO3a siRNA (siFOXO3a) and its negative control (si-NC) were designed by GenePharma Co., Ltd. (Shanghai, China). BV-2 cells (1.5 × 10^5^/well) in a 6-well plate were transfected with 200 pmol siFOXO3a and si-NC using Lipofectamine® 2000 (cat. no. 11668027, Invitrogen) according to the manufacturer's instructions. Transfected cells were incubated for an additional 24 h prior to OGD/R treatment. The corresponding sequences were as follows: siFOXO3a-sense 5′-GCUCUUGGUGGAUCAUCAATT-3′ and antisense 5′-TT CGAGAACCACCUAGUAGUU-3′ and siNC-sense 5′-UUCUCCGAACGUGUCACGUTT-3′ and antisense 5′-ACGUGACACGUUCGGAGAATT-3′. The efficiency of transfection was validated by comparing the level of FOXO3a between transfected and controlled cells by western blot.

### 2.12. Lactate Dehydrogenase (LDH) Activity Detection

The LDH content in the supernatants of SH-SY5Y cells was measured by using an LDH kit (cat. no. A020-2-2, Nanjing Jiancheng Bioengineering Institute, Nanjing, China) according to the manufacturer's manual. The absorbance OD value at 450 nm was measured by using a plate reader (ELx800, BioTek Instruments, Inc.). The LDH level of the control group was expressed as 100%, and the LDH levels of the other groups were normalized to this value.

### 2.13. Statistical Analysis

The data represent the mean ± standard deviation (SD) from at least three separate experiments. Statistical analyses were carried out using SPSS software version 15.0 (SPSS Inc., Chicago, IL). Student's *t*-test was used to analyze differences between two groups. When comparisons between multiple groups were carried out, one-way ANOVA followed by SNK tests was employed. Statistical significance was considered when *P* < 0.05.

## 3. Results

### 3.1. Isolation and Identification of MSC-exos

**Figure 1:**
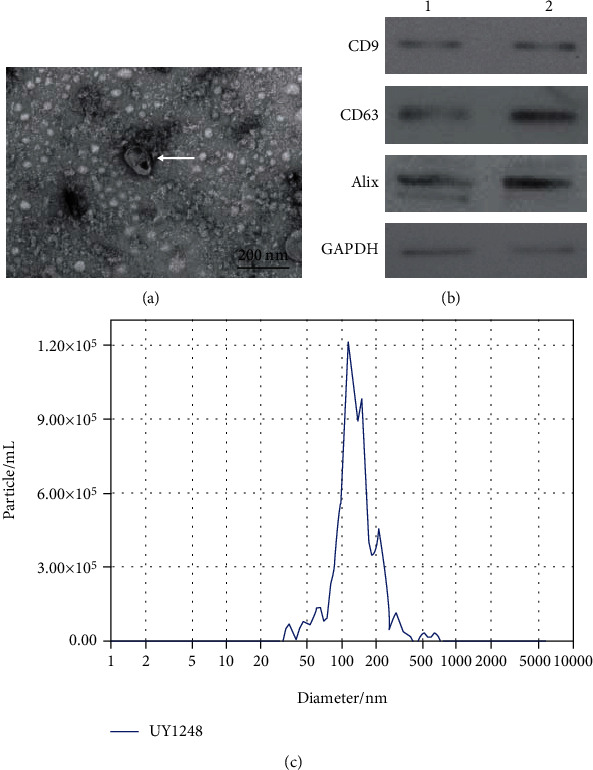
Identification of exosomes derived from human umbilical cord mesenchymal stem cells (MSC-exos). (a) Exosomes were observed under a transmission electron microscope. White arrow indicates the exosome. Scale bar: 200 nm. (b) Expression levels of exosomal markers (CD9, CD63, and Alix) were measured by western blotting from two samples (panels 1 and 2). (c) The size of exosomes was assessed by nanoparticle tracking analysis (NTA).

To investigate the potential roles of MSC-exos in I/R-induced neuronal injury, MSC-exos were first isolated and verified with transmission electron microscopy (TEM), western blot, and nanoparticle tracking analysis (NTA). The TEM results revealed that MSC-exos exhibited round-shaped morphology (Figure 1(a)), which is consistent with the typical exosomal morphology. Western blot results indicated that the levels of specific exosome surface markers (CD9, CD63, and Alix) were significantly upregulated in MSCs-exos (Figure 1(b)). NTA data indicated that the diameters of the MSC-exos were mostly approximately 100 nm (Figure 1(c)). Collectively, these results confirmed that the MSC-exos (30 *μ*g/mL medium) were successfully isolated and identified.

### 3.2. MSC-exos Attenuated OGD/R-Induced Pyroptosis of BV-2 Cells

**Figure 2:**
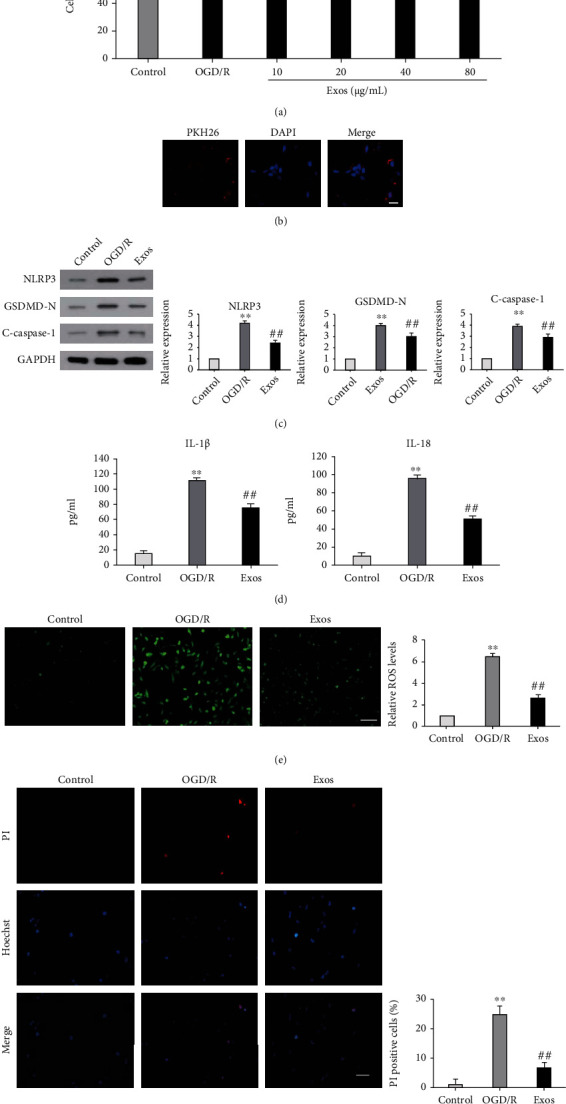
Effect of MSC-exos on OGD/R-induced pyroptosis of BV-2 cells. (a) BV-2 cells were cultured under three conditions: normal (control), OGD/R, and OGD/R with different concentrations of MSC-exos (Exos). After 24 h of culture, cell viability was determined by the CCK-8 assay. (b) MSC-exos are internalized by BV-2 cells. PKH26-labeled MSC-exos are shown in red, and DAPI-labeled nuclei are shown in blue. Scale bar: 50 *μ*m. (c–f) BV-2 cells were cultured under three conditions: normal (control), OGD/R, and OGD/R+40 *μ*g/mL MSC-exos (Exos). After 24 h of culture, the expression levels of GSDMD-N, cleaved caspase-1, and NLRP3 were detected by western blot (c), the levels of IL-1*β* and IL-18 were measured by ELISA with specific kits (d), and ROS levels were measured by staining the cells with dichlorofluorescein diacetate (CM2-DCFHDA). Scale bar: 100 *μ*m (e). Hoechst 33324/PI staining was measured by fluorescence microscopy. Scale bar: 100 *μ*m (f). ^∗∗^*P* < 0.01 versus the control group; ^##^*P* < 0.01 versus the OGD/R group; ^NS^*P* > 0.05 versus the 40 *μ*g/mL Exos group.

Previous studies demonstrated that microglial pyroptosis is an important cause of neuronal injury under I/R conditions [13, 14]. To investigate the effect of MSC-exos on OGD/R-induced pyroptosis, BV-2 cells were cultured in OGD conditions for 4 h and then cocultured with MSC-exos in the 24 h reperfusion phase. The results of the CCK-8 assay showed that OGD/R-induced viability of BV-2 cells was significantly decreased compared with that of the control group (Figure 2(a), *P* < 0.01); however, at concentrations of 10 to 80 *μ*g/mL, MSC-exo concentration dependently increased the viability of BV-2 cells following OGD/R injury (Figure 2(a), *P* < 0.01). No comparable improved effects on the viability of BV-2 cells were observed at concentrations of 40 *μ*g/mL and 80 *μ*g/mL (Figure 2(a), *P* > 0.05), and thus, a concentration of 40 *μ*g/mL was selected to conduct subsequent experiments. Immunofluorescence results showed PKH26-labeled MSC-exos (40 *μ*g/mL) in BV-2 cells, indicating successful uptake of MSC-exos (Figure 2(b)). The pyroptosis of BV-2 cells was then investigated by measuring the levels of pyroptosis-related proteins, including GSDMD-N, cleaved caspase-1, NLRP3, IL-1*β*, and IL-18, and intracellular ROS levels as well as Hoechst 33324/PI staining. After OGD/R exposure, the protein expression levels of GSDMD-N, cleaved caspase-1, and NLRP3, the levels of IL-1*β* and IL-18, intracellular ROS levels, and the percentage of PI-positive cells increased (Figures 2(c)–2(f), *P* < 0.01). In contrast, MSC-exo treatment partly abolished these effects of OGD/R exposure (Figures 2(c)–2(f), *P* < 0.01). Collectively, these data suggested that MSC-exos attenuated OGD/R-induced pyroptosis of BV-2 cells.

### 3.3. MSC-exos Attenuated SH-SY5Y Cell Injury Induced by OGD/R-Exposed BV-2 Cells

**Figure 3:**
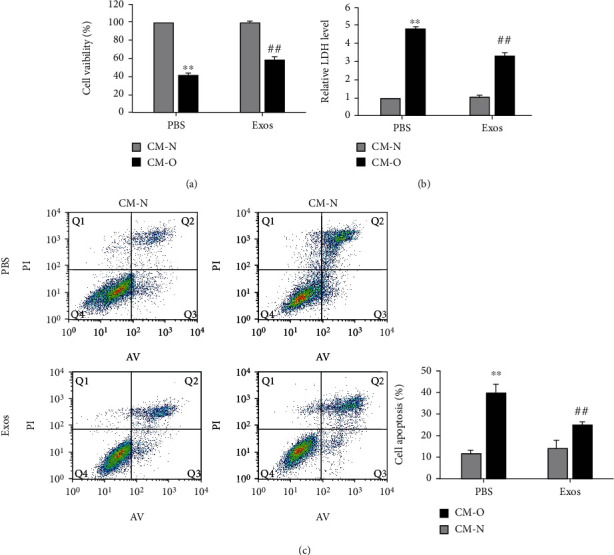
Effect of MSC-exos on injury to SH-SY5Y cells induced by OGD/R-exposed BV-2 cells. SH-SY5Y cells were cultured in cell-free conditioned medium (CM) collected from BV-2 cells with different treatments as follows: normal BV-2 cells treated with PBS (CM-N+PBS) or 40 *μ*g/mL MSC-exos (CM-N+Exos) and CM from OGD/R-exposed BV-2 cells treated with PBS (CM-O+PBS) or 40 *μ*g/mL MSC-exos (CM-O+Exos). After 24 h of culture, the viability of SH-SY5Y cells was determined by the CCK-8 assay (a), LDH release was measured by ELISA with a specific kit (b), and annexin V/propidium iodide (PI) staining was measured with a flow cytometer (c). ^∗∗^*P* < 0.01 versus the CM-N+PBS group; ^##^*P* < 0.01 versus the CM-O+PBS group.

To investigate whether MSC-exo treatment alleviates SH-SY5Y cell injury induced by OGD/R-exposed BV-2 cells, SH-SY5Y cells were exposed to cell-free conditioned medium (CM) collected from BV-2 cells with different treatments: normal BV-2 cells treated with PBS (CM-N+PBS) or 40 *μ*g/mL MSC-exos (CM-N+Exos) and OGD/R-exposed BV-2 cells treated with PBS (CM-O+PBS) or 40 *μ*g/mL MSC-exos (CM-O+Exos). SH-SY5Y cell injury was then measured by CCK-8, AV-FITC/PI, and LDH release assays. Our data showed that the CM-O+PBS group, compared to the CM-N+PBS group, displayed significantly decreased viability of SH-SY5Y cells (Figure 3(a), *P* < 0.01) and increased levels of LDH (Figure 3(b), *P* < 0.01) and percentage of apoptotic cells (Figure 3(c), *P* < 0.01). Compared with the CM-O+PBS group, the CM-O+Exos group showed significantly improved viability of SH-SY5Y cells (Figure 3(a), *P* < 0.01) and reduced levels of LDH (Figure 3(b), *P* < 0.01) and the percentage of apoptotic cells (Figure 3(c), *P* < 0.01). Collectively, these data indicated that MSC-exos attenuated SH-SY5Y cell injury induced by OGD/R-exposed BV-2 cells.

### 3.4. MSC-exos Prevented OGD/R-Induced Pyroptosis of BV-2 Cells by Enhancing Mitophagy

**Figure 4:**
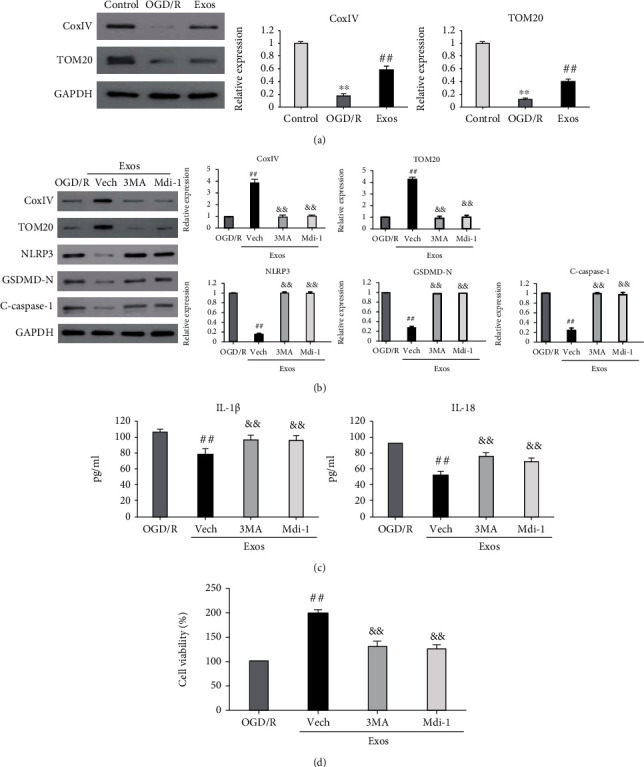
Mitophagy is required for the protective effect of MSC-exos against OGD/R-induced pyroptosis of BV-2 cells. (a) BV-2 cells were cultured under three conditions: normal (control), OGD/R, and OGD/R+40 *μ*g/mL MSC-exos (Exos). After 24 h of culture, the expression levels of TOM20 and COX IV were detected by western blot. (b–d) OGD/R-exposed BV-2 cells were treated as follows: untreated (OGD/R), MSC-exos treatment (Exos), pretreated with vehicle (Vech), 3-methyladenine (3MA, 10 mM), or mitochondrial division inhibitor-1 (Mdi-1, 10 *μ*M) followed by MSC-exos treatment. After 24 h of treatment, the expression levels of TOM20, COX IV, GSDMD-N, cleaved caspase-1, and NLRP3 were detected by western blot (b), the levels of IL-1*β* and IL-18 were measured by ELISA with specific kits (c), and cell viability was determined by the CCK-8 assay (d). ^∗∗^*P* < 0.01 versus the control group; ^##^*P* < 0.01 versus the OGD/R group; ^&&^*P* < 0.01 versus the Vech group.

Previous studies demonstrated that inhibition of mitophagy contributes to microglial pyroptosis [23, 29, 30]. Thus, we wondered whether mitophagy is involved in the protective effect of MSC-exos against OGD/R-induced microglial pyroptosis. As shown in Figure 4(a), OGD/R significantly downregulated the expression levels of mitophagy-related proteins, including translocase of outer mitochondrial membrane 20 (TOM20) and cytochrome c oxidase IV (COX IV), in BV-2 cells, which were clearly upregulated by MSC-exo treatment. When mitophagy was blocked with an autophagy inhibitor (3-methyladenine, 3-MA) or a mitophagy inhibitor (mitochondrial division inhibitor-1 (mdi-1)) in OGD/R-exposed BV-2 cells treated with MSC-exos, the expression levels of GSDMD-N, cleaved caspase-1, and NLRP3 were increased (Figure 4(b), *P* < 0.01), the levels of IL-1*β* and IL-18 were also significantly increased (Figure 4(c), *P* < 0.01), and the viability of BV-2 cells was significantly decreased (Figure 4(d), *P* < 0.01). Collectively, these data suggested that MSC-exos prevented OGD/R-induced pyroptosis of BV-2 cells by enhancing mitophagy.

### 3.5. MSC-exos Enhanced Mitophagy to Prevent OGD/R-Induced Pyroptosis of BV-2 Cells by Upregulating FOXO3a

**Figure 5:**
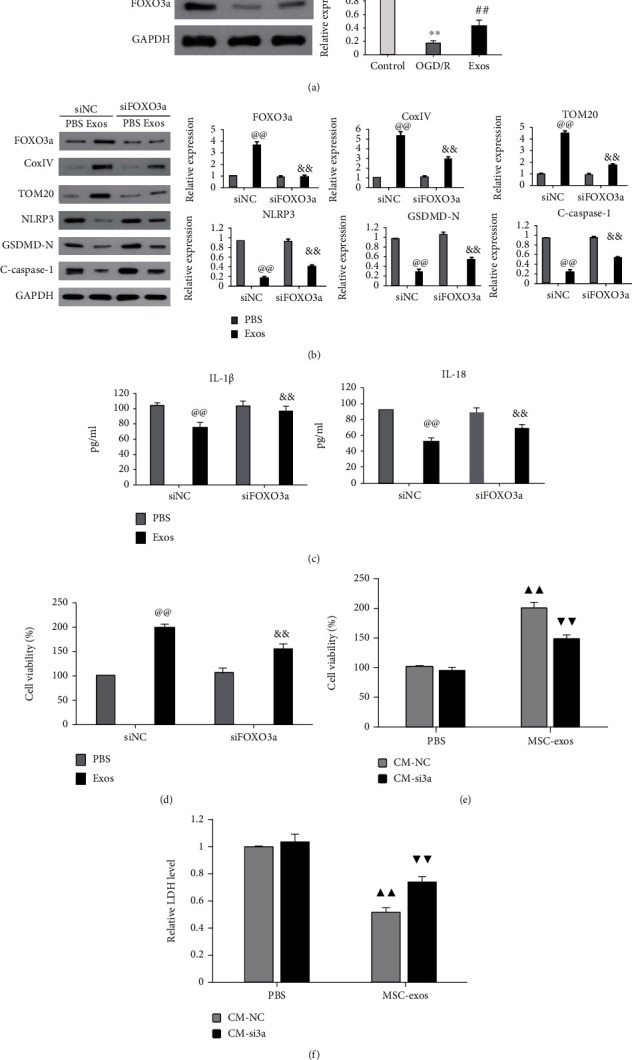
MSC-exos enhanced mitophagy to prevent OGD/R-induced pyroptosis of BV-2 cells by upregulating FOXO3a. (a) BV-2 cells were cultured under three conditions: normal (control), OGD/R, and OGD/R+40 *μ*g/mL MSC-exos (Exos). After 24 h of culture, the expression level of FOXO3a was detected by western blot. (b–d) BV-2 cells were transfected with negative control siRNA (siNC) or FOXO3a siRNA (siFOXO3a) and then subjected to OGD/R+PBS or OGD/R+MSC-exos (Exos) treatment. The expression levels of TOM20, Cox4IV, GSDMD-N, cleaved caspase-1, and NLRP3 were detected by western blot (b), the levels of IL-1*β* and IL-18 were measured by ELISA with specific kits (c), and cell viability was determined by the CCK-8 assay (d). (e, f) SH-SY5Y cells were cultured in CM collected from BV-2 cells treated as indicated in (b); after 24 h of culture, the viability of SH-SY5Y cells was determined by the CCK-8 assay (e), and LDH release was measured by ELISA with a specific kit (f). ^∗∗^*P* < 0.01 versus the control group; ^##^*P* < 0.01 versus the OGD/R group; ^@@^*P* < 0.01 versus the siNC+PBS group; ^&&^*P* < 0.01 versus the siNC+Exos group; ^▲▲^*P* < 0.01 versus the PBS+CM-NC group; ^▼▼^*P* < 0.01 versus the PBS+CM-si3a group.

FOXO3a has been demonstrated to play a key role in mitophagy and cell pyroptosis [31–34]. Therefore, we next investigated the involvement of FOXO3a in MSC-exo-induced mitophagy in our system. We first examined the change in FOXO3a expression level after MSC-exo treatment in OGD/R-exposed BV-2 cells. The results showed a significant decrease in FOXO3a expression under OGD/R conditions (Figure 5(a), *P* < 0.01). MSC-exo treatment significantly upregulated FOXO3a expression in OGD/R-exposed BV-2 cells (Figure 5(a), *P* < 0.01). The effects of FOXO3a upregulation on MSC-exo-induced mitophagy and pyroptosis inhibition were then investigated by transfecting BV-2 cells with FOXO3a siRNA (siFOXO3a). As shown in Figure 5(b), MSC-exo-induced FOXO3a upregulation was significantly inhibited by transfection with siFOXO3a. SiFOXO3a also significantly inhibited MSC-exo-induced upregulation of TOM20 and COX IV in OGD/R-exposed BV-2 cells (Figure 5(b), *P* < 0.01). Furthermore, the MSC-exo-induced increase in cell viability and decreased expression levels of GSDMD-N, cleaved caspase-1, and NLRP3 and levels of IL-1*β* and IL-18 in OGD/R-exposed BV-2 cells were significantly reversed by siFOXO3a (Figures 5(b)–5(d), *P* < 0.01). Collectively, these results indicated that upregulation of FOXO3a is required for MSC-exo-induced mitophagy and pyroptosis inhibition in OGD/R-exposed BV-2 cells.

We further determined whether FOXO3a is involved in the protective effect of MSC-exos against SH-SY5Y cell injury induced by OGD/R-exposed BV-2 cells. As shown in Figures 5(e) and 5(f), compared with CM from negative control siRNA transfection in OGD/R-exposed BV-2 cells (CM-siNC) treated with MSC-exos, CM from FOXO3a siRNA transfection in OGD/R-exposed BV-2 cells treated (CM-si3a) with MSC-exos significantly reduced the viability of SH-SY5Y cells (Figure 5(e), *P* < 0.01) and upregulated the release of LDH (Figure 5(f), *P* < 0.01). Taken together, these findings indicated that MSC-exos attenuated SH-SY5Y cell injury induced by OGD/R-exposed BV-2 cells by upregulating FOXO3a.

## 4. Discussion

Although Kabataş et al. clinically demonstrated the neural repair effect of MSCs in a patient with HIBD [4], the underlying mechanism remained largely unknown. MSC-exos were demonstrated to be one of the key mediators of MSC paracrine signaling and exerted a potential neuroprotective effect against hypoxic-ischemic- (H/I-) induced brain injury *in vitro* and *in vivo* [8, 9, 35, 36]. This suggested that MSC-exos may be more suitable for the treatment of HIBD than MSCs. However, the neuroprotective mechanism of MSC-exos has not been thoroughly elucidated. The present study investigated the potential protective mechanism of MSC-exos against I/R-induced neuronal injury. In the current study, TEM, NTA, and surface marker proteins confirmed that MSC-exos were successfully isolated and met international standards [37]. A further study showed that MSC-exos at a dose of 40 *μ*g/mL attenuated the injury of SH-SY5Y cells induced by OGD/R-exposed BV-2 cells. Our study was concordant with a previous study showing that MSC-exos could inhibit microglia-mediated neuroinflammation in perinatal brain injury [36].

Previous studies revealed that microglial pyroptosis plays an important role in the progression of neonatal HIBD [16]. Additionally, it has been shown that MSC-exos protect against H/I-induced injury by inhibiting pyroptosis in various cells, such as endothelial cells, cardiomyocytes, and neuronal cells [19, 35, 38, 39]. In the present study, MSC-exos significantly increased cell viability, inhibited the release of IL-1*β* and IL-18, and downregulated the expression levels of the key proteins associated with pyroptosis in OGD/R-exposed BV-2 cells. These findings suggested that the protective effect of MSC-exos against I/R-induced neuronal injury was associated with a reduced microglial pyroptosis. To our knowledge, the present study is the first to report that MSC-exos attenuate I/R-induced neuronal injury by preventing microglial pyroptosis.

We then investigated how MSC-exos inhibited microglial pyroptosis. Mitophagy is a process in which damaged mitochondria are selectively removed by autophagy mechanisms to maintain the stability of the intracellular environment [40]. Mitophagy has been shown to inhibit the activation of the NLRP3 inflammasome by reducing the release of ROS, which is an important activator of the NLRP3 inflammasome [41–43]. Recently, activation of mitophagy has been demonstrated to be a vital protective mechanism of MSC-exos against different diseases [24, 25]. Importantly, the inhibitory effect of mitophagy on pyroptosis has been demonstrated in several reports [23, 29, 30]. In accordance with these results, the present study showed that MSC-exos treatment significantly upregulated the levels of mitophagy-related proteins in OGD/R-exposed BV-2 cells. When mitophagy was blocked with 3-MA or mdi-1, the inhibitory effect of MSC-exos against OGD/R-induced pyroptosis was markedly reversed in BV-2 cells. These data strongly indicated that MSC-exos prevent OGD/R-induced pyroptosis of microglial BV-2 cells by enhancing mitophagy.

We also examined the potential activators of mitophagy in our system. FOXO3a, a transcription factor of the O subclass of the forkhead family, has been shown to promote the activation of mitophagy by regulating the expression levels of key proteins associated with mitophagy, such as Parkin and BNIP3 [31, 32]. FOXO3a has also been reported to inhibit pyroptosis by regulating the inflammatory response [33, 34]. Recently, FOXO3a was demonstrated to be an important downstream target of MSC-exos [17]. Our results showed that MSC-exos significantly upregulated FOXO3a expression in OGD/R-exposed BV-2 cells. FOXO3a siRNA not only reversed MSC-exo-induced mitophagy and pyroptosis inhibition in OGD/R-exposed BV-2 cells but also inhibited the protective effect of MSC-exos against BV-2 cell-mediated injury in SH-SY5Y cells. These findings suggested that MSC-exos ameliorate OGD/R-induced injury of SH-SY5Y cells by inhibiting pyroptosis of microglial BV-2 cells through promoting FOXO3a-dependent mitophagy. However, the mechanism by which FOXO3a regulates mitophagy in our system remains unclear. Mitophagy has been demonstrated to be mediated by two distinct pathways: one involves mitophagy receptors such as BNIP3, FUNDC1, and NIX and the other pathway is dependent on Parkin/PINK1 pathway-mediated ubiquitination [40]. Previous studies showed that FOXO3a could regulate Parkin expression at the transcriptional level [32, 44]. The expression level of BNIP3 was also reported to be regulated by FOXO3a [31, 45]. Whether the Parkin and/or BNIP3 pathway is involved in FOXO3a-mediated mitophagy in our system is an interesting point for us to investigate in the near future.

It is well known that MSC-exos contain various microRNAs (miRNAs), circular RNAs (circRNAs), long noncoding RNAs (lncRNAs), proteins, and mRNAs that can be stably transferred to recipient cells [46, 47]. Recent studies have shown that abnormally expressed circRNAs may be involved in the pathogenesis of neonatal HIBD [48]. Furthermore, exosome-shuttled circRNAs have been shown to improve ischemic brain injury [49]. Therefore, it is worthwhile to investigate which circRNAs in MSC-exos inhibit microglial pyroptosis and ameliorate neuronal damage by regulating FOXO3a in our system.

## 5. Conclusions

**Figure 6:**
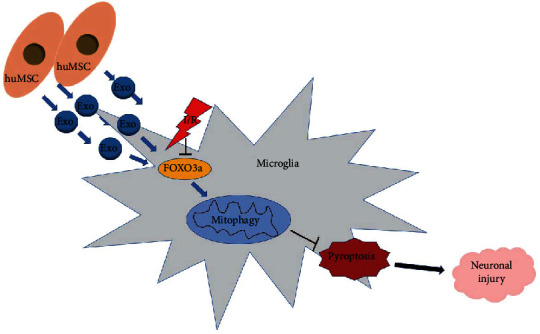
A schematic of depicting the mechanism of MSC-exos against microglia-mediated neuronal injury. MSC-exos increased FOXO3a expression to enhance mitophagy, therefore protecting microglia from I/R-induced pyroptosis and alleviating subsequent neuronal injury.

In conclusion, our findings revealed that MSC-exos upregulated FOXO3a expression to enhance mitophagy and repress I/R-induced pyroptosis in microglia, therefore alleviating subsequent neuronal injury (Figure 6). Although the effect and mechanism of MSC-exos against cerebral I/R injury found in the present study will be verified in further *in vivo* studies, the salient findings from the present study provide a microglia-centric view of the neuroprotective effect of MSC-exos. MSC-exos, as a new candidate therapeutic strategy, have excellent application prospects for neonatal HIBD.

## Data Availability

The data used to support the findings of this study are available from the corresponding author upon request.

## Abbreviations

MSC-exos: Mesenchymal stem cell-derived exosomes
HIBD: Hypoxic-ischemic brain damage
huMSCs: Human umbilical cord mesenchymal stem cells
WJ-MSCs: Wharton's jelly-derived mesenchymal stem cells
TEM: Transmission electron microscope
NTA: Nanoparticle tracking analysis
OGD/R: Oxygen-glucose deprivation/reoxygenation
I/R: Ischemia/reperfusion
CM: Conditioned medium
3-MA: 3-Methyladenine
Mdivi-1: Mitochondrial division inhibitor-1
CS: Cigarette smoke
TOM20: Translocase of outer mitochondrial membrane 20
COX IV: Cytochrome c oxidase IV
H/I: Hypoxic-ischemic.
